# Mediation Impact of Physical Literacy and Activity Between Psychological Distress and Life Satisfaction Among College Students During COVID-19 Pandemic

**DOI:** 10.1177/21582440231162503

**Published:** 2023-03-27

**Authors:** Xiaoxi Dong, Fan Huang, Xiangyun Shi, Menglin Xu, Zengyin Yan, Mehmet Türegün

**Affiliations:** 1 Chongqing University of Posts and Telecommunications, China; 2 University of Macau, China; 3 Nanjing University of Science and Technology, China; 4 Ohio State University, Columbus, USA; 5 Barry University, Miami, FL, USA

**Keywords:** physical literacy, physical activity behavior, life satisfaction, physical distress, SEM

## Abstract

The study aims to examine the mediation effects of physical literacy and physical activity behavior in a relationship between psychological distress and life satisfaction among Chinese college students during the real-life Coronavirus disease pandemic (COVID-19) circumstance. This study implemented a cross-sectional design, and 1,516 participants from 12 universities participated in this study. Structural equation modeling was used to examine a hypothesized model. The findings indicated an acceptable model fit (*X*^2^[61] = 508.2, Comparative Fit Index [CFI] = 0.958, Tucker Lewis Index [TLI] = 0.946, Root Mean Square Error of Approximation [RMSEA] = 0.076, 90% CI [0.070, 0.082], Standardized Root Mean Square Residual [SRMR] = 0.047). The results indicated that college students with low participation in physical activity could experience less than healthy living conditions. The findings offered empirical support to the theory that physical literacy could advance individuals’ healthy living by promoting physical activity participation. The study suggested that educational institutions and physical activity programs should cultivate individuals’ physical literacy in order to promote lifelong healthy living.

## Introduction

Physical inactivity (PI) is a significant contributor to noncommunicable diseases–cardiovascular diseases, various cancers, and chronic respiratory diseases–that have threatened public health around the world (WHO, 2002, 2010). Studies have indicated a gloomy trend in the worldwide prevalence of PI among the adult population in recent years (Guthold et al., 2008). According to Sjöström et al. (2006), 29% of the prevalence of sufficient physical activity (PA) was found across 15 European Union countries with a high to low range value of 44% to 23%. The study indicated that two-thirds of the population insufficiently participated in PA to provide the benefits of healthy living (Sjöström et al., 2006). Moreover, Guthold et al. (2008), who examined a worldwide prevalence of PI across 51countries mainly from low- and middle-income countries, indicated that 17.7% of the participants were described with characteristics of PI and the ratio of PI for women (19.8%) was higher than men (15.2%). Besides the working adult population, college students were also found to have high levels of PI. Studies have found that this population of students might cultivate an unhealthy lifestyle due to a challenging college life (Acharya et al., 2018; Pengpid et al., 2015). Pengpid et al. (2015) reported that 41.4% of undergraduate students had PI characteristics, with a range from 21.9% to 80.6% across 23 countries. Not only has the prevalence of PI increased around the world, but it has also extended the risk factors associated with PI to young adult populations. Furthermore, the prevalence of PI results in high costs for public healthcare systems (Ding et al., 2016; Katzmarzyk & Janssen, 2004).

The Coronavirus (COVID-19) pandemic has caused more than 1.5 million deaths and infecting more than 200 countries and territories (WHO, 2020). In an attempt to limit the spread of COVID-19, many countries have implemented semi-lockdown procedures and have issued social distancing guidelines resulting in people’s lives being compromised (Callow et al., 2020; He et al., 2021). Habitual engagement in PA is one of the compromises experienced among different populations across the world (Callow et al., 2020; He et al., 2021). According to Callow et al. (2020), based on the implementation of social distancing guidelines, almost 37.6% out of 1,046 older adults have experienced lower participation in PA in the United States and Canada. This study was consistent with a study conducted by Cheval et al. (2021), who reported that the time spent in PA by the adult population has decreased during the COVID-19 induced lockdown period in France and Switzerland. Qin et al. (2020) also reported that 57.5% out of 12,107 adults insufficiently participated in PA during home quarantine in China. Moreover, He et al. (2021) found that the mean of vigorous- and moderate-intensity PA minutes per day showed a statistically significant decrease from before to during the semi-lockdown among the Chinese population.

Physical literacy (PL) has been adopted as a goal of physical education for promoting a healthy lifestyle among individuals (Chen, 2015; Roetert & MacDonald, 2015). According to Whitehead (2010), PL does not only devote itself to promoting active engagement in physical activity, but it also cultivates an attitude of lifelong healthy living. PL presents a holistic educational concept that comprises various domains (Caldwell et al., 2020; Corbin, 2016). The affective domain consists of motivation and confidence, emphasizing a positive attitude toward physical activity (Dudley, 2015; Edwards et al., 2017). In addition, the affective domain is an essential feature that encourages individuals to become actively engaged in physical activity. Furthermore, the competence domain concentrates on an interactional relationship between human movements and their environments (Jurbala, 2015; Whitehead, 2001). According to Dudley (2015), the interactional relationship can help individuals to develop meaningful physical movements in order to participate in various physical activities. Likewise, the cognitive domain comprises two critical elements—one is toward a healthy lifestyle, and the other is toward physical performance (Edwards et al., 2017; Whitehead, 2010). According to Ennis (2015), knowledge is fundamental support for helping individuals to conceptualize how to react to different situations in their lives. The behavioral domain focuses on building up individuals to take responsibility for participating in lifelong physical activity regularly to improve well-being (Yi et al., 2020). All domains of PL complement one another for helping individuals to reflect on their lifelong progress with PL. Studies have provided empirical evidence to support that PL is positively associated with physical activity and health-promoting behavior among different populations, including school-aged children and college students, in different countries (Caldwell et al., 2020; Kwan et al., 2019; Ma et al., 2020).

**Figure 1.**
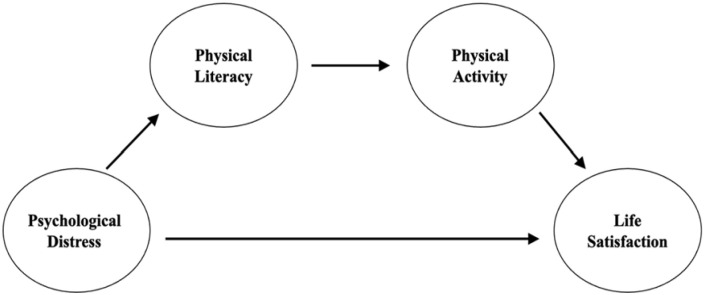
The hypothesized structural equation modeling.

College students, who are a vulnerable population in the early life transition stages (Acharya et al., 2018), have returned to their universities for in-person classes in the Fall semester of 2020 in China. Special attention needs to examine college students’ psychological well-being and habitual engagement in PA in order to promote their healthy living during the continued COVID-19 circumstances. Psychological well-being is a process of self-evaluation of personal life experiences and perceptions of wellness (Bryant & Veroff, 1982; Diener et al., 1999). Further, psychological well-being consists of multiple dimensions, that is, positive and negative affect, self-acceptance, symptoms of distress, and life satisfaction (Bryant & Veroff, 1982; Ryff & Keyes, 1995; Ryff & Singer, 2008). In addition, life satisfaction is a significant element of well-being (Diener et al., 1999). Studies indicated that PA is a valuable strategy to promote psychological well-being while decreasing psychological distress (PD) (Blair et al., 2004; Warburton et al., 2006). Moreover, PD conceptually consists of negative impacts of stress, depression, and anxiety that negatively influence individuals’ well-being (Diener et al., 1999; Ryff et al., 2006). Theories and studies have indicated that potentially PL can improve individuals’ wellness by promoting PA participation (Cairney et al., 2019; Kwan et al., 2019; Ma et al., 2020). However, limited studies have investigated a simultaneous relationship among PL, PA behavior, and psychological well-being among college students. According to recommendations for moderate-intensity and vigorous-intensity aerobic PA for promoting health by WHO (2010), this study aims to investigate the mediation impact of PL and moderate- and vigorous-intensity PA time per day between psychological distress and life satisfaction (LS) among Chinese college students during the real-life COVID-19 circumstances. Five alternative hypotheses are presented below, and the hypothesized structural equation modeling is displayed in Figure 1.

H_1_: Psychological distress is statistically significantly and negatively associated with life satisfaction.
H_2_: Psychological distress is statistically significantly and negatively associated with physical literacy.
H_3_: Physical literacy is statistically significantly and positively associated with physical activity.
H_4_: Physical activity is statistically significantly and positively associated with life satisfaction.
H_5_: Physical literacy and physical activity are statistically significant mediators in the relationship between psychological distress and life satisfaction.

## Methods

### Participants

This study adopted a cross-sectional design. Convenience sampling was implemented, and the recruitment of participants took place at 12 universities located in six cities in mainland China. In addition, the 12 universities compromised seven public universities and five private universities. A total of 1,516 Chinese college students (female = 871 and male = 645) with a mean age of 19.65 (*SD* = 1.49) participated in this study. In addition, the majors of the participants were varied, that is, education, medicine, sport, business, nursing, and science and technology. The demographic information of the participants reported for the six cities was as follows: Xi ’an (one university with *N* = 39), Bei Jing (one university with *N* = 46), Tian Jin (one university with *N* = 90), Huai Nan (one university with *N* = 36), Chong Qing (two universities with *N* = 205), and Nan Jing (six universities with *N* = 1,100). Given confidentiality concerns, an Informed Consent form was obtained from all participants.

### Measures

The *Satisfaction with Life Scale* (SWLS) is a 7-point Likert-type scale ranging from strongly disagree to strongly agree with five items evaluating the level of individuals’ life satisfaction (Diener et al., 1985). This study implemented the SWLS instrument to evaluate Chinese college students’ life satisfaction. The result of internal consistency for the SWLS instrument indicated a high internal consistency (Cronbach’s α = .92).

The *Perceived Physical Literacy Instrument* (PPLI) is a 5-point Likert scale ranging from strongly disagree to strongly agree with three items each for three subscales: the sense of self and self-confidence, self-expression and communication with others, and knowledge and understanding (Sum et al., 2016). The PPLI instrument was adapted to examine Chinese college students’ PL. Good internal consistencies of the PPLI instrument of the study were found for the subscales of sense of self and self-confidence (Cronbach’s α = .83), self-expression and communication with others (Cronbach’s α = .77) and knowledge and understanding (Cronbach’s α = .78).

The *Depression Anxiety Stress Scale – 21 Items* (DASS-21) is a 4-point Likert-type scale with responses ranging from did not apply to me at all to apply to me very much with seven items each for three subscales–depression, anxiety, and stress (Lovibond & Lovibond, 1995). The DASS-21 instrument was adopted to examine the level of depression, anxiety, and stress of college students. Additionally, the DASS-21 instrument indicated good internal consistencies for the three subscales: depression (Cronbach’s α = .83), anxiety (Cronbach’s α = .80), and stress (Cronbach’s α = .82).

The *International Physical Activity Questionnaire Short Form* (IPAQ-SF) was adopted to examine the amount of moderate-intensity and vigorous-intensity PA during the past 7 days (International Physical Activity Questionnaire [IPAQ], 2019). The IPAQ-SF has been indicated as a reliable instrument to evaluate the amount of moderate-intensity and vigorous-intensity PA during the past 7 days (Macfarlane et al., 2007). Four items from the IPAQ-SF were used to collect Chinese college students’ daily moderate- and vigorous-intensity PA time over 7 days.

### Procedure

After the Institutional Review Board approved this research, the researchers contacted 12 faculty members who have worked for universities in China with a request to cooperate in recruiting participants at their universities. The 12 faculty members have worked in six different cities in mainland China: one faculty member each for Bei Jing, Tian Jin, Xi’an, and Huai Nan, two in Chong Qing, and six in Nan Jing. After the members consented to collaborate in the data collection procedures, an online survey link was sent to them. The potential participants could then access the online survey with a link forwarded by their faculty members. An informed consent form was placed on the first page of the survey for participants to read and give consent. After granting consent, the potential participants could then continue to answer the survey questions. The period of collecting data was between September 1 and December 10, 2020.

### Data Analysis

The study investigated the mediation effects of physical literacy and physical activity behavior on psychological distress and life satisfaction in real-life contexts among college students. Structural equation modeling (SEM) was used to examine the hypothesized model. Additionally, for evaluating the model fit, the cut-off values for the goodness-of-fit indices consisted of the value of the Comparative Fit Index (CFI) and Tucker Lewis Index (TLI) greater than 0.90 and the value of Standardized Root Mean Square Residual (SRMR) and Root Mean Square Error of Approximation (RMSEA) smaller than 0.80, suggested by Hu and Bentler (1999) and Schumacker and Lomax (2010). The R package *lavaan*, developed by Rosseel (2012), was used to evaluate the hypotheses. A statistical significance was determined as an alpha of .05.

## Findings

### Summary Statistics

The descriptive statistics were conducted and displayed in Table 1. The mean of vigorous-intensity PA for the total sample of 1,516 was 5.29 min per day (*SD* = 6.07) with a range from 0 to 21.4 min per day. Further, 615 out of 1,516 participants reported not participating in any vigorous-intensity PA during the last week. The mean of moderate-intensity PA for the total sample of 1,516 was 7.48 min per day (*SD* = 8.39) with a range from 0 to 32.1. 523 out of 1,516 reported not participating in moderate-intensity PA during the last week. Pearson’s correlation analysis was conducted to examine the correlations between manifest variables (MVs) loaded for PL, PA, PD, and LS. In addition, the variable of age represented demographic information. The results illustrated that all MVs were statistically significantly correlated except MVs of vigorous-intensity PA minutes per day with ls5 and MVs of stress and moderate-intensity PA minutes per day with anxiety and stress. Moreover, age was statistically significantly and negatively correlated to vigorous-intensity and moderate-intensity PA minutes per day, respectively.

**Table 1.** Descriptive Statistics and Correlation Matrix (*N* = 1,516).

	vpd	mpd	ls1	ls2	ls3	ls4	ls5	kut	set	ssct	dept	anxt	strt	age
vpd	-													
mpd	0.38***	-												
ls1	0.11***	0.11***	-											
ls2	0.09***	0.07**	0.84***	-										
ls3	0.07**	0.06*	0.85***	0.89***	-									
ls4	0.07*	0.09***	0.67***	0.70***	0.72***	-								
ls5	0.03	0.06*	0.55***	0.56***	0.56***	0.58***	-							
kut	0.12***	0.13***	0.38***	0.38***	0.37***	0.33***	0.20***	-						
set	0.16***	0.13***	0.47***	0.50***	0.48***	0.45***	0.39***	0.50***	-					
ssct	0.17***	0.17***	0.46***	0.46***	0.44***	0.42***	0.35***	0.62***	0.66***	-				
dept	−0.07**	−0.09***	−0.36***	−0.36***	−0.38***	−0.33***	−0.22***	−0.29***	−0.35***	−0.29***	-			
anxt	−0.05*	−0.05	−0.26***	−0.27***	−0.29***	−0.27***	−0.21***	−0.23***	−0.32***	−0.28***	0.77***	-		
strt	−0.03	−0.05	−0.31***	−0.34***	−0.34***	−0.32***	−0.29***	−0.20***	−0.35***	−0.30***	0.77***	0.80***	-	
age	−0.22***	−0.19***	−0.04	−0.03	−0.04	−0.01	0.01	−0.05	0.06*	0.06*	0.06*	−0.01	0.00	-
*M*	5.29	7.48	4.66	4.64	4.68	4.53	3.76	11.87	9.91	9.81	3.65	4.18	5.20	19.65
*SD*	6.07	8.39	1.36	1.39	1.38	1.51	1.77	2.28	2.49	2.62	3.42	3.30	3.87	1.48
skew	0.98	1.14	−0.19	−0.15	−0.20	−0.17	0.13	−0.55	0.13	0.02	0.78	0.69	0.46	0.43
kurt	−0.10	0.55	0.01	−0.16	−0.14	−0.38	−0.85	0.37	−0.12	−0.17	−0.29	−0.16	−0.43	−0.34
0 min	*N* = 615 (40.6%)	*N* = 523 (34.5%)												
<10 min	*N* = 588 (38.8%)	*N* = 530 (35%)												
<20 min	*N* = 281 (18.5%)	*N* = 317 (20.9%)												
<35 min	*N* = 32 (2.1%)	*N* = 146 (9.6%)												

*Note.* vpd = vigorous-intensity physical activity minutes per day; mpd = moderate-intensity physical activity minutes per day; ku = knowledge and understanding; ssc = sense of self and sense of confidence; *se* = self-expression and communication with others; skew = skewness; kurt = kurtosis; ls = life satisfaction; ls included five items, from ls1 to ls5. The same hereinafter.

* *p* < .05. **p < .01. ***p < .001.

### Structural Equation Modeling Evaluation

SEM was used to examine the hypothesized model of the mediation impacts of PL and PA between PD and LS. Maximum likelihood estimator with robust (MLR) was used as suggested by Rosseel (2020) because of the concern of violation of normality assumption, as some values of skewness and kurtosis were close to an absolute value of 1. Moreover, the latent variable (LV) of PA was loaded by vigorous- and moderate-intensity PA time per day, and the LV of PL was loaded by the total scores of the three subscales. Furthermore, LV of PD was loaded by the total scores of the three subscales of stress, depression, and anxiety. The LV of life satisfaction was loaded by its five items. The result of the structural model illustrated an acceptable model fit (*X*^2^[61] = 508.2, CFI = 0.958, TLI = 0.946, RMSEA = 0.076, 90% CI [0.070, 0.082], SRMR = 0.047). The loadings of the MVs were statistically significant (*p* < .001) to their respective LVs.

**Figure 2.**
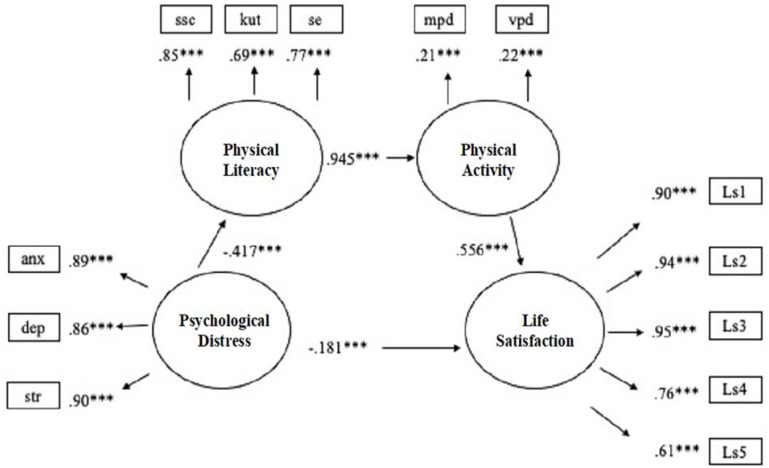
The standardized coefficients of the mediation model.

Four statistically significant and direct path coefficients and one statistically significant and indirect path coefficient were found in the structural model. The LV of PD was statistically significantly and negatively associated with the LVs of PL and LS, and the LV of PL was statistically significantly and positively associated with the LV of PA. Meanwhile, the PA was statistically significantly and positively associated with LS. Moreover, the LVs of PL and PA were statistically significant mediators in the relationship between PD and LS, as displayed in Table 2 and Figure 2. Furthermore, the *R*^2^ value for the three endogenous LVs of LS, PA, and PL was 0.421, 0.892, and 0.173, which indicated that the 42.1%, 89.2%, and 17.3% of the variations in LVs of LS, PA, and PL could be explained by the structural model simultaneously. Based on the effect size index suggested by Cohen (1988), *R*^2^ value for the LVs of LS and PA indicated a large effect size, and the LV of PL had a medium effect size. The structural model consisted of a statistically significant partial-mediation effect. The LV of PD was statistically significantly and indirectly impact of the LV of LS mediated by PL and PA (*b* = −0.091, 95% CI [−0.109, −0.073], *SE* = 0.009, *t* = −9.957, *p* < .001). Furthermore, regarding the sum of the indirect effect and the direct path of PD on LS, a total statistically significant effect was found (*b* = −0.167, 95% CI [−0.192, −0.142], *SE* = 0.013, *t* = −13.128, *p* < .001).

**Table 2.** Structural Model Path Coefficients.

LVs	*b*	β	95% CI	*SE*	*t*
pd – ls	−.075	−.181	[–.099, –.051]	0.012	−6.140***
pd – pl	−.319	−.417	[–.366, –.271]	0.024	−13.212***
pl – pa	0.566	.945	[0.402, 0.730]	0.084	6.772***
pa – ls	0.506	.556	[0.333, 0.679]	0.088	5.735***

*Note*. pd = psychological distress; pl = physical literacy; pa = physical activity; *b* = unstandardized coefficient; β = standardized coefficient; 95% CI = 95% confidence interval. The same hereinafter.

* *p* < .05. **p < .01. ***p < .001.

## Discussion

This study investigated the mediation impacts of PL and PA on a relationship between PD and LS. Four statistically significant direct associations and one statistically significant indirect association were found in the structural model. The results of the study indicated an acceptable model fit (*X*^2^[61] = 508.2, CFI = 0.958, TLI = 0.946, RMSEA = 0.076, 90% CI [0.070, 0.082], SRMR = 0.047). The structural model could explain 42.1%, 17.3%, and 89.2% of the variations in LVs of LS, PL, and PA simultaneously.

First, the finding indicated a lack of engagement in vigorous- and moderate-intensity PA among Chinese college students. The results of this study indicated that means of the moderate- and vigorous-intensity PA of this study were 7.48 and 5.29 min per day. The results were roughly similar to the study conducted by Ma et al. (2020), who reported that the means of the moderate- and vigorous-intensity PA was 7.34 and 4.94 min per day for a study conducted among a population of Chinese undergraduate students aged 18 to 21 years. The result of the current study for the PA time per day was also consistent with the study conducted by He et al. (2021). He et al. (2021) reported that the mean vigorous-intensity or moderate-intensity PA time per day was 5.4 min (*SD* = 2 min) for females and 3.2 min (*SD* = 3.2 min) for males during the semi-lockdown time induced by the presence of COVID-19 in China in 2020. The current study might indicate that college students inadequately participated in PA for promoting healthy living during the semi-lockdown time in relation to the COVID-19 circumstance. A possible reason is that college students might have limited options to access sports facilities and socialize with others in-person to participate in outdoor activities because college students needed to follow the social distancing guidelines during the COVID-19 condition. Therefore, college students have had fewer opportunities to engage in PA. Further, the vigorous- and moderate-intensity PA time per day was statistically significantly and negatively associated with age, which indicated a reduction of PA participation associated with an increase in age among the participants. The finding illustrated the prevalence of physical inactivity patterns reported by Guthold et al. (2008) that older people were more likely to be physically inactive. Moreover, the finding was consistent with a study conducted by Pengpid et al. (2015), finding that increasing age among college students was positively associated with physical inactivity. The present study offers evidence to enrich studies evaluating the prevalence of PA among the Chinese college student population who participated in PA insufficiently during COVID-19 conditions.

The LV of PL was statistically significantly and positively associated with LV of PA loaded by vigorous- and moderate-intensity PA time per day. The result indicated that an increase in PL was associated with an increase in PA participation. The result was consistent with studies conducted by Kwan et al. (2019) and Ma et al. (2020), who found a positive relationship between PL and engagement in PA among college students. Choi et al. (2018) also reported that PL had a positive effect on participation in PA among the adolescent population. The present study offers statistical evidence to support the theoretical framework of PL conceptualized by Whitehead (2010), that PL is beneficial for promoting a level of participation in PA among college students. Specifically, this study indicated that cultivating college students’ positive affective and cognitive domains of PL could ultimately advance the degree of PA participation based on the LV of PL indicators. Further, the LV of PA was statistically significantly and positively associated with the LV of LS. The result was consistent with a study conducted by Maher et al. (2015), finding that those who had a higher level of PA participation daily had a higher level of LS in adulthood. In addition, the authors found a significant but weak and positive correlation between PA and LS that was consistent with the present study. The present study was also consistent with a study reported by Pengpid and Peltzer (2019), who found a positive association between moderate-intensity PA and LS with a sample of college students across 24 countries. Based on the empirical results, promoting daily engagement in PA could be beneficial for encouraging college students’ LS.

Furthermore, studies have reported that college students experienced a higher level of PD (Acharya et al., 2018; Wu et al., 2015). The present study indicated statistically significant and negative associations between PD and LS and PD and PL, which indicated those who experienced a higher level of PD had a lower level of LS and PL. Based on the results of this study, there were statistically significant mediation effects of PL and PA in the relationship between PD and LS. The result supported the theory conceptualized by Cairney et al. (2019) that PL can positively impact psychological well-being by promoting habitual engagement in PA. Specifically, the structural model illustrated an indirect path that a higher level of PL could advance a higher level of daily PA, which could ultimately foster a higher level of LS. In addition, the indirect path could alleviate the negative impact of PD on LS. The findings also provided statistical evidence to bolster PL theory proposed by Whitehead (2010) that not only does PL promote a level of engagement in PA, but it also cultivates a lifelong healthy living attitude among individuals.

## Implications

Physical Education is an indispensable component in the holistic development of human beings, and Quality Physical Education is an essential entry point to cultivating 21st-century skills, promoting inclusion for everyone, participating and understanding values such as cooperation, partnership, teamwork, discipline, peace, and work. This evidence-based research emphasizes the importance of physical education and provides empirical support to the theory that physical literacy could advance individuals’ healthy living by promoting physical activity participation.

The study suggested that educational institutions and physical activity programs should cultivate individuals’ physical literacy to promote lifelong healthy living. To address the issue of physical inactivity, we recommend curriculum reforms to include sufficient time and opportunities for students to participate in various physical activities. Due to the pandemic situation and online learning, when we are restricted in space and movements, it is even more important to stay active and healthy. Different kinds of indoor activities, for example, yoga, Taichi, and aerobics, could be introduced to students to stimulate their interest in sports and provide more PA choices.

Previous evidence showed that financial support for physical education was minimal, and it affected the quality of service that should have been provided. For example:

Government austerity measures in many Commonwealth countries since the 1980s have increasingly removed public sector support to physical education in schools and recreational sport and physical activity in communities. Freely available sport has been replaced by various pay-to-play initiatives by the public and private sectors and nongovernmental organisations. The latter, including sport for development (SfD) organisations, supported by sponsorships, corporate social responsibility and charitable donations, have moved in to provide cheaper alternatives to comprehensive state programmes. These alternatives may well benefit those they reach but they cover only a fraction of the population and are rarely sustained. Many rely primarily on well-meaning but inexperienced youthful volunteers (Commonwealth Secretariat, 2020).

The government needs to involve several stakeholders, including finance, education, health, youth and sport, planning, and transportation, to mention but a few as the inactivity problem needs to be embraced by several players. Now, people need to be prepared and professionals should be the ones directing the process and being accompanied by the government sector. Countries should fulfill international agreements and show respect with more evidence (e.g., Kazan Action Plan). Policies in place need to be revisited and the future depends on who can make cooperative work in their societies to have a more active PA society. Education sets habits, but the policies in place need to reinforce them and communities need to have the possibility to develop their practice in their local places.

The WHO, (2020) has provided guidelines for physical activity and sedentary behavior. Organizations and particularly the school sector should be aware of the indications of this document to make it possible to push forward and make visible the relevance of being active. The recommendation goes to people of all ages to become more active and provides clear indications on how to do it. Future research to investigate how different gender identities could impact the mediation effects of physical literacy and physical activity behavior in a relationship between psychological distress and life satisfaction is recommended.

## Limitations and Conclusion

One of the limitations is that the findings of this study might not be generalized to other populations because this study used a convenience sampling methodology, which might raise a concern with sampling error. In addition, in-depth insights might not be gained from survey research. Observations and interviews could be added to provide supplementary information for this study. The 1,516 students who participated in this study may not be representative of the total number of college students in China. Due to different cultural, economic, and political backgrounds, the findings of the current study must be read in this context. Hence, it cannot be generalized to the entire college student population in China and other countries. An additional limitation is that the latent loadings of PA were relatively low, which might be due to self-reported information. Participants self-reported their activities and perceptions. Therefore, the self-reported activities might potentially not be objective about their activities. Likewise, the finding cannot yield causality because of the cross-sectional research design. The authors recommend future studies might employ an experimental design to examine potential causal effects among the LVs.

This study might be the first study to examine the mediation effects of PL and PA in a relationship between PD and LS during COVID-19 conditions. The results indicate that college students with low participation in PA could experience less than healthy living conditions during the COVID-19 pandemic. Therefore, the study recommended that educational institutions should pay special attention to increasing college students’ PA participation to promote college students’ wellness during the COVID-19 pandemic. Furthermore, the study illustrated that PL and PA were significant mediators that might promote LS while alleviating PD simultaneously. The findings offered empirical support to the theory that PL could advance individuals’ healthy living by promoting PA participation. This study suggested that educational institutions and physical activity programs should cultivate college students’ PL and PA participation in order to foster their wellness.
